# Characterization of pilot-scale dilute acid pretreatment performance using deacetylated corn stover

**DOI:** 10.1186/1754-6834-7-23

**Published:** 2014-02-18

**Authors:** Joseph Shekiro III, Erik M Kuhn, Nicholas J Nagle, Melvin P Tucker, Richard T Elander, Daniel J Schell

**Affiliations:** 1 National Bioenergy Center, National Renewable Energy Laboratory, 617 Cole Blvd, 80401 Golden, CO, USA

**Keywords:** Deacetylated corn stover, Lignocellulosic pretreatment, Dilute acid, Xylose, Pilot

## Abstract

**Background:**

Dilute acid pretreatment is a promising process technology for the deconstruction of low-lignin lignocellulosic biomass, capable of producing high yields of hemicellulosic sugars and enhancing enzymatic yields of glucose as part of a biomass-to-biofuels process. However, while it has been extensively studied, most work has historically been conducted at relatively high acid concentrations of 1 - 4% (weight/weight). Reducing the effective acid loading in pretreatment has the potential to reduce chemical costs both for pretreatment and subsequent neutralization. Additionally, if acid loadings are sufficiently low, capital requirements associated with reactor construction may be significantly reduced due to the relaxation of requirements for exotic alloys. Despite these benefits, past efforts have had difficulty obtaining high process yields at low acid loadings without supplementation of additional unit operations, such as mechanical refining.

**Results:**

Recently, we optimized the dilute acid pretreatment of deacetylated corn stover at low acid loadings in a 1-ton per day horizontal pretreatment reactor. This effort included more than 25 pilot-scale pretreatment experiments executed at reactor temperatures ranging from 150 – 170°C, residence times of 10 – 20 minutes and hydrolyzer sulfuric acid concentrations between 0.15 – 0.30% (weight/weight). In addition to characterizing the process yields achieved across the reaction space, the optimization identified a pretreatment reaction condition that achieved total xylose yields from pretreatment of 73.5% ± 1.5% with greater than 97% xylan component balance closure across a series of five runs at the same condition. Feedstock reactivity at this reaction condition after bench-scale high solids enzymatic hydrolysis was 77%, prior to the inclusion of any additional conversion that may occur during subsequent fermentation.

**Conclusions:**

This study effectively characterized a range of pretreatment reaction conditions using deacetylated corn stover at low acid loadings and identified an optimum reaction condition was selected and used in a series of integrated pilot scale cellulosic ethanol production campaigns. Additionally, several issues exist to be considered in future pretreatment experiments in continuous reactor systems, including the formation of char within the reactor, as well as practical issues with feeding herbaceous feedstock into pressurized systems.

## Background

Conversion of lignocellulosic biomass to fuels and chemicals continues to be a focus of worldwide research. While there are many different conversion pathways for producing biofuels, the biochemical conversion of biomass to ethanol is an area of keen focus. This pathway generally involves the hydrolysis of the polysaccharide fraction of biomass to produce sugars followed by fermentation of these sugars to ethanol. Typically, a pretreatment process is employed that alters the biomass physical structure and chemical composition making the residual polysaccharides more susceptible to conversion to monomeric sugars by enzymatic hydrolysis. Pretreatment with dilute sulfuric acid also hydrolyzes hemicellulose to monomeric and oligomeric sugars. The primary goal when optimizing pretreatment performance is to identify operating conditions that maximize production of sugars from all available polysaccharides by varying parameters such as temperature, reaction time, and catalyst concentration. As such, most pretreatment researchers now report sugar yields from enzymatic hydrolysis of the pretreated solids as a key metric for determining optimum pretreatment conditions.

Recent reviews of pretreatment technologies are available from various authors, and an entire issue of the journal, *Bioresource Technology,* is devoted to the evaluation of different pretreatment technologies on the same biomass feedstock [1-4]. These results are summarized in a later publication [5]. The introductory article to the *Bioresource Technology* issue, as well as the review paper cited above, describe the outcomes of an effective pretreatment process [6]. For example, a pretreatment process should increase the effective surface area, decrease cellulose crystallinity, and increase pore size of the pretreated biomass. These changes improve enzymatic digestibility of the hemicellulose and cellulose remaining in the solids. Effective pretreatment techniques have been developed using acids, alkalis, hot water, steam explosion, oxidizers, ammonia, solvents, and ionic liquids. Many approaches rely on combined physical and chemical modifications to achieve good biomass pretreatment. Once optimized, most pretreatment processes are able to produce solids with good enzymatic digestibility, as noted by Elander *et al*. [5].

Many pretreatment studies are performed in small, batch-type reactors. While these reactors are sufficient for the characterizing pretreatment performance across a wide range of operating conditions, the optimum conditions found in small batch reactors do not translate well to pilot-scale reactors due to differences in heat and mass transfer characteristics. There are also physical modifications to the biomass that occur when it is fed into continuous, high-pressure reactors. The impact of these physical modifications of the biomass, including particle size reduction, on pretreatment performance is not well understood. For example, biomass is fed to many continuous pilot-scale reactors by a pressurized screw feeder, also known as a plug screw feeder. This device imparts high levels of compression and shear forces on the biomass, and is generally believed to modify the particle size distribution and other structural characteristics of the feedstock, but it is not known what impact these modifications have on pretreatment performance. Nevertheless, pilot-scale reactors likely produce pretreated materials similar to those produced in commercial-sized reactors, so evaluating downstream performance on these materials is vital to advancing process understanding.

In this work, we employed a newly constructed 20 to 40 dry kg/h continuous pilot-scale pretreatment reactor system to pretreat biomass using dilute sulfuric acid. The system consists of a pressurized screw feeder that feeds biomass into a pressurized horizontal-tube reactor containing a series of screw augers for conveying the biomass and controlling residence time. Pretreated biomass is discharged through two full-port ball valves into a live-bottom flash tank. There are similarities to the National Renewable Energy Laboratory’s (NREL’s) older pilot-scale pretreatment system, but there are also significant differences, including lower electrical power requirements, horizontal reactor orientation instead of vertical, and a more effective discharge system [7]. This new reactor system was designed to be highly versatile, capable of pretreatment using acid or base chemistry and an array of biomass feedstocks. The reactor comprises several horizontal tubes, which can be reconfigured to achieve residence times between 2 minutes and 2 hours. In general, horizontal reactors are believed to offer improved residence time control over vertical reactor systems. However, vertical reactor systems are simpler and less expensive, both of which are advantages at larger scales [8].

Several researchers throughout the world have recently reported work in continuous pilot-scale pretreatment systems. Horizontal-tube reactors are currently being used in Italy, Denmark, Canada, and Taiwan for hydrothermal or acidic pretreatment of various biomass types at feed capacities ranging from 100 to 200 dry kg/h [9-13]. Commercial companies are operating other pilot-scale pretreatment systems throughout the world, but equipment details and performance results on these systems are not readily available [14].

The goal of this work was to assess performance of dilute sulfuric acid pretreatment of deacetylated corn stover in our new continuous pilot-scale pretreatment reactor at various operating conditions. The intent was to identify the best pretreatment operating conditions using low acid concentrations, less than 0.5% (w/w) sulfuric acid concentration at reaction conditions, prior to executing a series of integrated pilot-scale campaigns for the production of ethanol. The feedstock for this work was deacetylated corn stover produced by treating raw corn stover with dilute sodium hydroxide prior to pretreatment. The work of Chen *et al*. demonstrated that dilute acid pretreatment of deacetylated corn stover produced higher monomeric xylose yields and improved cellulose digestibility of the pretreated solids compared to corn stover that was not deacetylated [15]. In the current study, performance was characterized by measuring pretreatment conversion yields and enzymatic cellulose digestibility of the pretreated solids.

## Results and discussion

### Characterization of pretreatment performance

To evaluate performance across a wide range of pretreatment severities, a series of 25 runs were performed in the pilot-scale pretreatment reactor at various operating conditions using a single lot of deacetylated corn stover. The operating conditions spanned pretreatment temperatures of 150 to 170°C and residence times of 10 to 20 minutes with sulfuric acid concentrations of 0.14 to 0.30% (w/w) in the reactor. The residence time was estimated based on screw rotational speed and may not be the true residence time. The pretreated corn stover (PCS) at 27 to 35% (w/w) total solids (TS) contained liquid phase monomeric xylose and glucose concentrations ranging from 36.7 to 94.1 g/L and 0.5 to 13.7 g/L, respectively, depending on pretreatment severity. Oligomeric xylose and glucose concentrations ranged from 4.9 to 20 g/L and 0.0 to 6.1 g/L, respectively. In single-stage dilute acid pretreatment, optimized for the production of monomeric and/or oligomeric xylose, the cellulose fraction of the biomass remains generally intact, with conversion of cellulose to glucose of up to 6% [16]. In this study, the total glucose (monomeric and oligomeric) yield in pretreatment was between 3.5 and 6.1% of the glucan present in the original corn stover. Complete concentration and yield data can be found in Additional file 1.

Mass balance closure results were calculated in several ways to assess process performance and data validity. Gravimetric mass closure was calculated by measuring mass of TS fed to the pretreatment reactor and accounting for all mass in the process effluents as either insoluble or dissolved solids in the PCS, or as organic compounds present in the flash or reactor vent vapor-streams. The calculation procedure is available in the methods section of this manuscript. Gravimetric mass balance closure for each run ranged from 100 to 102%, indicating good tracking of mass entering and exiting the system Additionally, total and component (e.g. glucan, xylan) carbon mass balances around the pretreatment reactor system were performed as discussed in the methods section. Total carbon balance closure ranged from 95 to 102%, with no discernible trend within the range of pretreatment severities tested. The good overall mass closure results suggest the accurate measurements of mass flow rates were made and that any component-level mass balance closure discrepancies may be attributed to analytical error and lack of quantification of specific compounds.

**Figure 1:**
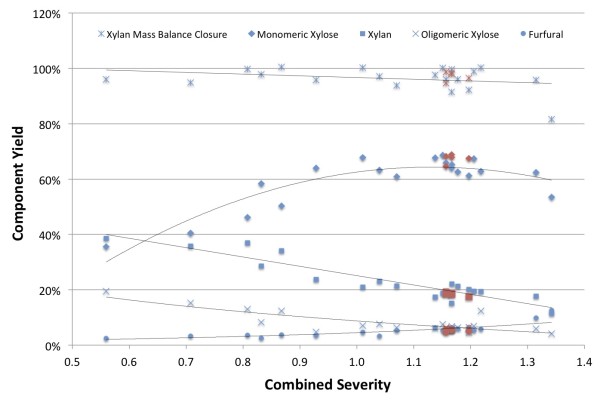
**Xylan derived component yields from dilute acid hydrolysis of deacetylated corn stover as a function of pretreatment severity.** Red points represent replicated runs conducted at 160°C, 10 minutes with 0.34% (w/w) H_2_SO_4_ in the pretreatment reactor. Trends are shown by regressed lines.

Component carbon mass balances for glucan and xylan ranged from 95 to 107% and 92 to 100%, respectively. Xylan balance closures were observed to decrease beyond a combined severity of 1.25 as shown in Figure 1. This may be attributed to degradation of furfural to compounds that were not directly quantified. Conversely, these compounds are likely to be detected as lignin, as soluble sugars and other extractives react to form solids, which interfere with common insoluble lignin measurement techniques [17,18]. The mass balance closures for each individual experiment are included in Additional file 1.

The yields of compounds derived from xylan hydrolysis are depicted in Figure 1 plotted by combined severity. Combined severity (log R_0_) is an expression of pretreatment intensity as a function of reaction time (t, minutes), temperature (T,°C) and pH, and is defined as [19]:

(1) logR0=logt×eT−10014.75−pH

Monomeric xylose yields increased from a combined severity of 0.56 to approximately 1.00 to 1.15, where it reached a broad peak of approximately 68%. The limited amount of data beyond a combined severity of 1.3 suggest monomeric xylose yields were beginning to decrease. The proportion of original xylan remaining in the insoluble solids decreased linearly within the range of test conditions from approximately 40% to just over 10% at the highest severity condition tested. Furfural yields increased linearly from 2.5% to about 6% between combined severities of 0.56 and 1.25, with a sharper increase after a combined severity of 1.25, reaching 12.6% at a combined severity of 1.34. As expected, the yield of xylo-oligosaccharides fell with increasing severity, because higher pretreatment severity converts more oligomers to monomers.

Several studies exploring pilot-scale dilute acid pretreatment of corn stover found optimum monomeric xylose yields at combined severities of 1.2 to 1.6, with corresponding monomeric xylose yields of approximately 70% and total xylose yields of up to 80% [7,16,20]. However, because different researchers employ different equipment and methodologies, a comparison of our results to other studies can be difficult. For example, the actual residence time in pilot-scale continuous reactors may be different to the theoretical calculation because of back-mixing and non-ideal flow. Our preliminary work measuring the residence time distribution in the reactor used in this study is suggesting the actual residence time may as much 50 to 100% greater than the calculated value (data not shown). Thus, the true combined severity of experiments conducted in this study may be 10 to 25% greater than the values presented here.

To assess process variability, five runs were executed at pretreatment conditions of 10 minutes, 160°C, and 0.26% (w/w) sulfuric acid in the reactor. The average and standard deviation of the TS content of the PCS for these runs was 30.9% ± 3.3%. Average monomeric and oligomer xylose concentrations were 83.6 ± 8.1 g/L and 7.6 ± 1.3 g/L, respectively. Average component yields for the various xylan derived fractions were 67.4% ± 1.7% for monomeric xylose, 6.1% ± 0.4% for oligomeric xylose, 5.3% ± 0.4% for furfural, and 18.5% ± 0.6% for unreacted xylan. The average xylan mass balance closure was 97.3% ± 1.7%. Potential sources of variation include cyclical deviation in stream flows (for example, steam, corn stover, water) caused by feedback control loops and corresponding changes in operating conditions as well as variability in the moisture content of corn stover. All of these factors lead to slight differences in reaction pH and temperature and ultimately differing pretreatment efficacies. Reaction pH, combined severity and effective acid concentration for each pretreatment experiment are available in Additional file 1.

**Figure 2:**
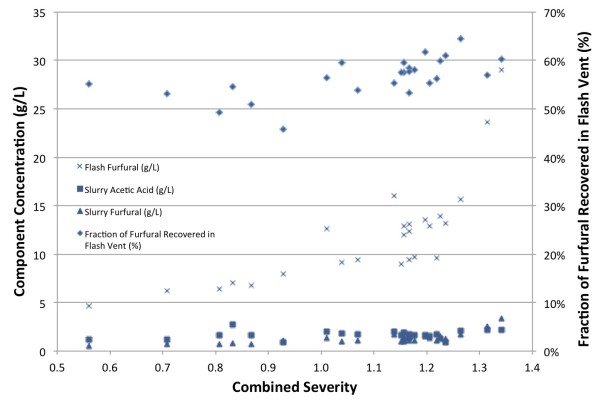
Pretreated corn stover liquor acetic acid and furfural concentrations and furfural concentration in the flash vapor, along with fraction of the total furfural recovered in the flash vapor.

Pretreated biomass exits the pretreatment reactor through a pair of alternating full-port ball valves into an atmospheric pressure flash tank separating into a flash vapor stream and a PCS stream. The flash evaporation process releases volatile compounds from the PCS slurry and the most easily measured components are furfural and acetic acid. The concentrations of these components in the PCS liquor fraction and furfural concentration in the condensed flash vapor are plotted as functions of combined severity in Figure 2. Concentrations of furfural in the flash vapor and PCS liquor fraction increase with increasing furfural production associated with increasing pretreatment severity (see Figure 1). Approximately 50 to 60% of the furfural produced is recovered in the flash vapor stream, reaching concentrations in this stream near 30 g/L at the highest pretreatment severity. The more important consideration is furfural concentration in the PCS liquor, as furfural is a potent fermentation inhibitor [17,18]. Below a combined severity of 1.25 the furfural concentrations were less than 1.5 g/L, a concentration tolerated by many microorganisms. At the highest pretreatment severity, the furfural concentration was near 4.0 g/L, which is inhibitory to many microorganisms.

Because the deacetylation process extracts approximately 80% of acetate from the corn stover, the acetic acid concentrations in the liquor are independent of pretreatment severity, a finding also observed by Chen *et al*. [21]. Consequently, acetic acid concentrations were only 1.5 to 2.5 g/L, drastically lower than the 8 to 12 g/L observed in the literature for high-solids dilute acid pretreatments [22,23]. Only 5 to 10% of acetic acid produced during pretreatment was recovered in the flash vent stream.

### Feedstock reactivity

**Figure 3:**
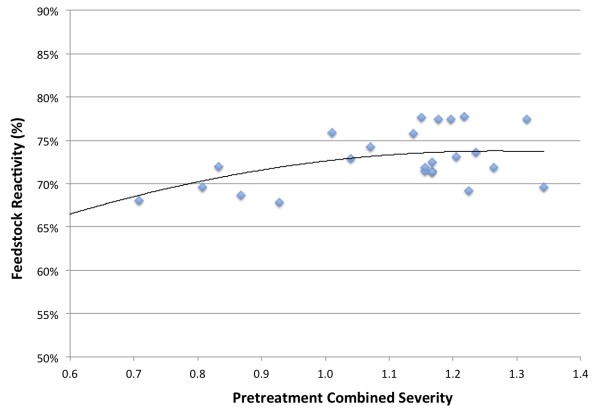
Feedstock reactivity (based on total monomeric and oligomeric xylose as defined in the methods section) as a function of combined severity.

As the primary carbohydrate in corn stover is glucan, xylan-to-xylose yield in isolation is not an effective metric for assessing pretreatment performance. Rather, the feedstock reactivity, a relative measure of the total sugar yield was calculated from the total xylan-to-xylose yield and the yield of monomeric glucose from enzymatic cellulose hydrolysis of each PCS sample. The calculation is described in the methods section. Calculated feedstock reactivity for each of the experiments is shown in Figure 3.

The yield of monomeric glucose from cellulose during enzymatic hydrolysis slowly increased with increasing pretreatment combined severity (results not shown but are available in Additional file 1). This behavior is typically ascribed to increasing accessibility of the cellulose to enzymatic attack as a greater fraction of the hemicellulose is solubilized with increasing pretreatment severity [7,16]. However, within the range of pretreatment severities tested, feedstock reactivity appeared to reach a broad maximum of 70 to 80% at the higher combined severities. Even though xylose yields were decreasing after a combined severity of 1.15, increasing cellulose digestibility was causing the feedstock reactivity to remain relatively constant at combined severities greater than 1.1. Previous work using dilute-acid pretreated slurry produced at reduced acid loadings in pilot reactor systems achieved lower enzymatic cellulose to glucose conversions (60 to 70%) than the present work (70 to 80%) [24]. These experiments achieved significantly lower xylan-to-xylose yields in pretreatment.

While the conversion of xylo-oligomers and insoluble xylan to xylose during enzymatic hydrolysis was not evaluated in these experiments, previous studies have shown that a significant proportion of the oligomers can be converted enzymatically to xylose [25-27]. This finding was confirmed in the subsequent integrated pilot-scale ethanol production campaigns previously mentioned (data not shown). Based on these results, the pretreatment condition selected for the pilot-scale campaigns was 160°C, 10 minutes, and a reactor acid concentration of 0.34%. This condition balanced the need to preserve xylan for potential subsequent conversion, while also maximizing xylose yield from pretreatment and glucose yield from enzymatic hydrolysis.

### Operational observations

During commissioning the pretreatment reactor, two issues arose that are worthy of discussion. The first issue was intermittent difficulty feeding deacetylated corn stover into the reactor using the plug screw feeder. It was determined though trial and error that 1.3 to 1.9 cm (0.50 to 0.75 in) hammer-milled corn stover had a higher quantity of fine particles and lower cohesive strength than knife-milled corn stover. Consequently, we believe that as the corn stover is driven forward and compressed by the plug screw feeder, the material within the rotating flights separates from the fraction packed within the anti-rotation device, causing the forward movement of corn stover to slow or halt. This separation, commonly described as plug shearing, results in cessation of feeding and is manifested as a discrete decrease in the electrical current required by the plug screw feeder motor and an accumulation of corn stover in the inlet zone of the plug screw feeder. If not quickly addressed, a sheared plug can result in rapid pressure release from the reactor back through the plug screw feeder. Figures of several anti-rotation mechanisms are not reproduced here, but are available in many current patents [28-30]. It should be noted that this problem is not seen during feeding of either hammer-milled or knife-milled untreated (raw) corn stover.

**Figure 4:**
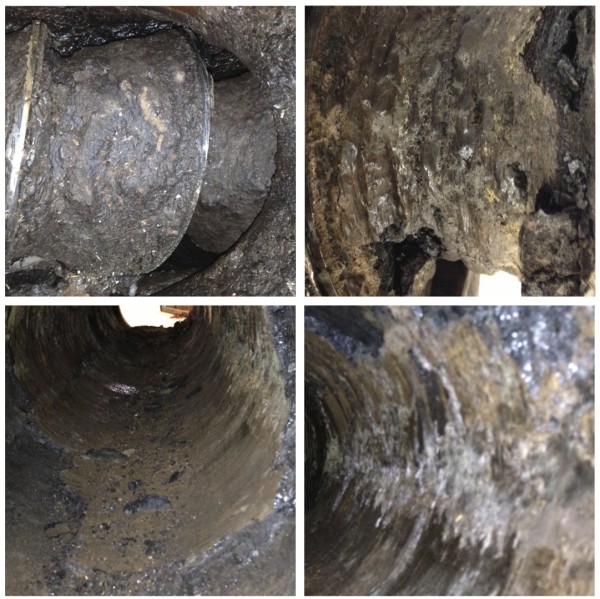
Images of carbonaceous deposits observed on reactor augers and walls.

Additionally, after approximately 200 hours of operation, significant accumulation of a black, coke-like substance was observed on the walls of the reactor and within the flights of the augers. This substance was also found within the pressurized but non-jacketed discharge sections of the reactor. The material, shown in Figure 4, was generally brittle, but insoluble in acid or base solutions even at elevated temperatures. To clean the reactor it was completely disassembled and surfaces were scraped to remove as much of the material as possible. An elemental analysis of the material found it to be approximately 66% carbon, 27% oxygen, 5% hydrogen and 1% nitrogen by mass, with a C:O ratio of roughly 2.2:1. This material was significantly enriched in carbon content relative to native corn stover, which has a C:O ratio of approximately 1:1. The composition is consistent the condensation products that can form when furans, such as furfural, react with monomeric and oligomeric sugars to form insoluble humins [31].

## Conclusions

The intent of this work was to understand pretreatment of deacetylated corn stover at low acid concentrations in pilot-scale reactors. This is the first known work testing pretreatment of deacetylated biomass in pilot-scale reactors for fuel production. Low acid concentrations are desirable because they may eliminate the need for exotic materials of construction for pretreatment reactors. For example, lower-cost stainless steels could be used in place of expensive Hastelloy. Additionally, a lesser quantity of caustic reagent is needed for neutralizing these slurries prior to enzymatic hydrolysis, which reduces chemical costs and perhaps downstream operating cost, particularly for wastewater treatment.

Commissioning the newly installed horizontal reactor system identified several operational challenges that should be addressed in similar reactor designs, or considered in ongoing pretreatment operations. First, the structural characteristics of a feedstock must be evaluated in addition to its composition and recalcitrance. It is clear that some feedstocks, or methods of preparing feedstock create a non-ideal substrate for pressurized (plug) screw feeder operation. This work highlighted the need for maintenance and cleaning programs to prevent or minimize the deposition of charred solids on reactor surfaces.

Deacetylated corn stover was successfully pretreated and an optimum pretreatment condition of 160°C, 10 minutes, and 0.26% H_2_SO_4_ (combined severity of 1.15 to 1.20) was identified for future work. This condition balanced the need to minimize the formation of degradation products, maximize sugar yields, and reduce acid consumption. Pretreated slurries produced in this severity range achieved total xylose concentrations of over 100 g/L at 30% TS and feedstock reactivity (an estimate of total sugar yield) up to 77%. Furfural and acetic acid concentrations ranged between 1.3 to 1.5 g/L and 1.0 to 1.9 g/L respectively, and should be well-tolerated by most fermentative microorganisms.

Given that the range of operating conditions explored in this study is still somewhat limited, opportunities still exist for further performance improvements. However, integrated pretreatment, enzymatic hydrolysis and fermentation performance data are needed to optimize overall process performance.

## Methods

### Feedstock

The corn stover was harvested in Emmetsburg, IA, USA in October 2010 and shipped to Idaho National Laboratory (INL, Idaho Falls, ID, USA). There it was processed using the pilot-scale feedstock-handling unit operated by INL as described by Yancey *et al*. and hammer-milled to pass through a 1.9-cm- (0.75-in)-round rejection screen [32]. The composition of the raw feedstock is described in Table 1.

**Table 1:** Composition of raw corn stover and alkaline extracted and acid impregnated corn stover

	**Sucrose (%)**	**Glucan (%)**	**Xylan (%)**	**Galactan (%)**	**Arabinan (%)**	**Lignin (%)**	**Protein (%)**	**Ash (%)**	**Acetyl (%)**	**Water/ethanol-extractible others (%)**	**Total mass closure (%)**
**Raw corn stover**	0.2 ± 0.1	35.1 ± 0.6	23.3 ± 1.6	1.9 ± 0.1	3.5 ± 0.2	15.1 ± 0.3	2.1 ± 0.2	8.5 ± 0.4	1.9 ± 0.2	5.8 ± 1.5	97.6 ± 0.6
**Deacetylated corn stover**	ND	44.7 ± 0.9	25.3 ± 0.7	1.7 ± 0.1	3.7 ± 0.2	16.3 ± 1.3	0.2 ± 0.4	3.8 ± 0.3	0.3 ± 0.2	ND	96.0 ± 0.8

Standard deviations calculated using duplicate analyses of six independent samples of raw corn stover and 21 independent samples of deacetylated and acid-impregnated corn stover. ND – None detected.

### Feedstock deacetylation and acid impregnation

**Figure 5:**
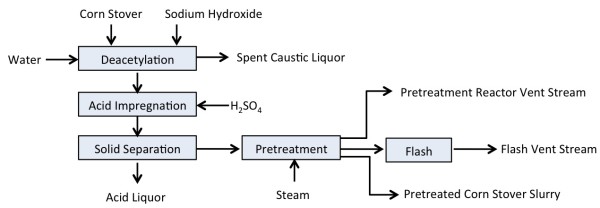
Block flow diagram of the deacetylation, impregnation and pretreatment processes.

The overall process flow for this work is shown in Figure 5. Corn stover deacetylation and sulfuric acid impregnation was performed in a 1,900-L horizontal jacketed vessel (American Process Systems, Gurnee, IL, USA) agitated with paddles suspended from a central shaft. Dry corn stover (100 to 120 dry kg) was added to the tank along with a dilute sodium hydroxide solution (0.4% w/w) to achieve a TS loading of 8% (w/w). The slurry was continuously mixed, heated to 80°C and held for 2 hours. The free-liquid fraction was subsequently allowed to drain overnight. The solids were then re-suspended in water for one hour at 10% TS and allowed to drain once again. A dilute sulfuric acid solution (0.5% or 0.8% w/w) was then added to the drained solids to achieve a TS loading of 8% (w/w). After thoroughly mixing the acid-impregnated solids at room temperature for 2 hours, the slurry was pumped to a continuous screw press (Vincent Corp. Model CP10, Tampa, FL, USA) to dewater the solids to 45 to 50% (w/w) TS. The deacetylated and acid-impregnated corn stover (DAICS) solids were collected in drums and stored at room temperature prior to use in pretreatment. Samples of DAICS were taken from each batch prepared as described above and the average composition of the 25 samples is shown in Table 1. However, yield calculations for each experiment were performed using the composition of the individual batch of DAICS used for that experiment.

### Pilot-scale pretreatment

The horizontal pretreatment reactor (Mesto Inc., Norcross, GA, USA) was operated at temperatures of 150 to 170°C and residence times of 10 to 20 minutes. The residence time was calculated based on the screw speed, assuming no back-mixing of the solids. A full list of the pretreatment conditions used in this study is shown in Additional file 1. The pretreatment reactor was preheated using steam jackets for 1 hour and allowed to reach the desired temperature before adding DAICS into the reactor. DAICS was manually fed to an Acrison Inc. feeder (Moonachie, HJ, USA), which delivered feedstock at a constant volumetric rate to a weigh-belt (K-tron, Pitman, NJ, USA) configured to deliver a constant mass flow rate of biomass into the pretreatment system at 25 to 30 dry kg/h. After discharge from the weigh- belt, the DAICS was fed into the pressurized zone of the reactor by pressurized (plug) screw feeder and heated to the desired temperature by direct steam injection. DAICS was conveyed through the horizontal tubes by a screw auger with residence time controlled by the rotational speeds of the screw. The pretreated slurry was discharged into an atmospheric pressure flash tank through a pair of automated, air-actuated ball valves opening and closing in an alternating pattern so as to prevent rapid depressurization of the reactor.

Upon entering the flash tank, the hydrolyzate separates into a pretreated high-solids (approximately 30% w/w) slurry stream (PCS), and a flash-tank vent stream. The flash-tank vent stream was condensed in a heat exchanger. The flash vapor flow-rate was determined from mass and energy balance calculations around the pretreatment process. Additionally, volatile compounds were removed from the pretreatment reactor by venting a small vapor stream (pretreatment reactor vent) from the high point of the reactor at a flow rate of 5 kg/h. The flow rate was measured by a coriolis flow meter (Emerson Controls (Micro Motion), Boulder, CO, USA). This stream was also condensed in a heat exchanger.

At each pretreatment operating condition, PCS was collected in a drum for a period of 20 minutes; samples of DAICS, PCS and condensates from both the flash and reactor vents were taken and stored at 4°C prior to compositional analysis. After all samples were collected, the pretreatment reactor set points were changed for the next experiment. Upon a change in reactor set point such as steam pressure, residence time, or feed rate, or other process disruption, at least two residence times were allowed to elapse after all flow rates reached steady state prior to the collection of any samples. Data such as stream flows and reactor conditions were collected in real time and recorded by a data acquisition and control system.

The gravimetric mass balance was calculated by dividing the sum of mass flow rates of solids recovered in each of the process effluent streams (PCS, reactor vent, flash vent) by the mass flow rate of dry corn stover fed to the reactor, as measured by the weigh-belt. The calculation was performed using mass flow rate measurements (Ṁ, kg/h), TS (% w/w), densities (ρ, kg/L), and concentrations (C, g/L) of various compounds in each stream, as shown in Equations 2 to 6:

(2) $${\overset{˙}{M}}_{\text{Feedstock}}={\overset{˙}{M}}_{\text{Feedstock},\mathrm{in}}\;x\%{\mathrm{TS}}_{\text{Feedstock}}$$

(3) $${\overset{˙}{M}}_{\text{PCS}}={\overset{˙}{M}}_{\text{PCS},\text{Out}}\;x\%{\mathrm{TS}}_{\text{PCS}}$$

(4) $${\overset{˙}{M}}_{\;\text{Reactor}\;\text{Vent}}=\frac{{\overset{˙}{M}}_{\text{Reactor}\;\text{Vent}}}{\rho_{\text{Reactor}\;\text{Vent}}}\;x\;\left(C_{\text{Furfural}}+C_{\text{Acetic}\;\text{Acid}}\right)$$

(5) $${\overset{˙}{M}}_{\;\text{Flash}\;\text{Vent}}=\frac{{\overset{˙}{M}}_{\text{Flash}\;\text{Vent}}}{\rho_{\text{Flash}\;\text{Vent}}}\;x\;\left(C_{\text{Furfural}}+C_{\text{Acetic}\;\text{Acid}}\right)$$

(6) $$\begin{array}{l} \text{Gravimetric}\;\text{mass}\;\text{balance}\;\text{closure}\;\left(\%\right)\\ \;=\frac{{\overset{˙}{M}}_{\text{PCS}}+{\overset{˙}{M}}_{\;\text{Reactor}\;\text{Vent}}+{\overset{˙}{M}}_{\;\text{Flash}\;\text{Vent}}}{{\overset{˙}{M}}_{\text{Feedstock}}} \end{array}$$

In addition to the gravimetric mass balance, process yields and component carbon mass balances were calculated by a carbon mass balance technique using a method previously described by Hatzis *et al*. [7,33]. For calculating xylan and glucan mass balances, it was assumed that all furfural was derived from only xylan, and that 5-hydroxy-methyl furfural (HMF) was only derived from sucrose.

### Cellulose digestibility assay

Cellulose digestibility is defined as the percent of the cellulose in the PCS converted to monomeric glucose during enzymatic hydrolysis at the conditions discussed below. PCS was neutralized with 14% (w/w) ammonium hydroxide at room temperature (22°C) to approximately pH 5. Sufficient neutralized PCS, enzyme (Cellic CTec 2, Novozymes, Davis, CA, USA), and 50 mM citrate buffer (pH 4.9) solution (total mass of 100 g) was added to 250-mL capped bottles to achieve a 20% PCS TS loading. The enzyme loading was 40 mg protein/g cellulose with the assumption that the cellulose content of each PCS sample was 50% (w/w) on a dry basis. As the protein loading was quite high and the range of pretreatment severities fairly narrow, it was assumed that small differences in actual enzyme loading would not be detectable. The bottles were placed in a shaking incubator rotating at 150 rpm and maintained at 48°C. Enzymatic hydrolysis was performed for 96 h. The difference between final and initial monomeric glucose concentration was used to calculate cellulose digestibility (monomeric glucose yield) according to the method described by Roche *et al*. [34]. Each analysis was performed in duplicate and average values are reported.

Using cellulose digestibility and the total (monomers and oligomers) xylose yield from pretreatment, a parameter called the feedstock reactivity (FR) was calculated as shown in Equation 9, where X is the mass fraction of a given component. The parameter is a simplified representation of sugar produced by combined pretreatment and enzymatic hydrolysis and was used for making relative performance comparisons.

(7) $$\mathrm{FR}=\frac{\text{Yield}\;\mathrm{of}\;{\text{Glucose}}_{\mathrm{EH}}\times X_{\text{Glucan},\text{Feedstock}}+\text{Yield}\;\mathrm{of}\;\text{Total}\;{\text{Xylose}}_{\mathrm{PT}}\times X_{\text{Xylan},\text{Feedstock}}}{X_{\text{Glucan},\text{Feedstock}}+X_{\text{Xylan},\text{Feedstock}}}$$

### Chemical analysis

Sugar concentrations were measured by HPLC using an Agilent 1100 series HPLC (Santa Clara, CA, USA) with a Shodex SP0810 carbohydrate column (Shawa Denko K.K., Kawasaki, Japan) and a de-ashing guard cartridge (BioRad Laboratories, Hercules, CA, USA). The column temperature was 85°C and the mobile phase was ultra-pure water at a flow rate of 0.6 mL/minute. Acetic acid, HMF, and furfural concentrations were measured by HPLC using a Phenomenex Rezex RFQ Fast Fruit H + organic acid column and Cation H + guard cartridge (BioRad Laboratories) at 55°C. The mobile phase was dilute sulfuric acid (0.01 N) at a flow rate of 0.6 mL/minute. A refractive index detector was used for compound detection for both columns. Mixed component standards were periodically run with the HPLC samples to verify calibration accuracy. The density of liquid samples was measured using an Anton-Parr model DMA-500 density meter (Anton Parr USA, Inc., Ashland, VA, USA).

The composition of pretreated solids was determined using a two-stage acid digestion procedure [35]. Concentrations of total soluble sugars in pretreated liquor samples were determined using a mild dilute acid hydrolysis procedure and HPLC analysis [36]. Concentration of the oligomeric sugar was the difference between total and monomeric sugar concentration. Slurry TS concentrations were determined by drying samples at 45°C in a vacuum oven (0.6 bar) until repeated weight measurements were constant. Slurry insoluble solid concentrations were determined by a six-step washing and centrifugation procedure [7]. Triplicate measurements were performed on each sample.

The enzyme was desalted prior to measuring the protein content. Desalting was performed on a HiPrep 26/10 desalting column (GE Healthcare, Uppsala, Sweden) with a Sephadex G-25 column matrix using a 2-mL sample. The mobile phase was a 50 mM Tris, 150 mM NaCl pH 5 buffer at a flow rate of 10 mL/minute. Protein concentration was measured using the Pierce BCA (BCA Protein Assay Kit, Pierce, IL, USA) assay following the manufacturer’s protocols with bovine serum albumin as the protein standard. The measurement was performed in triplicate.

## Abbreviations

DAICS: deacetylated and acid-impregnated corn stover; FR: feedstock reactivity; HMF: 5-hydroxy-methyl furfural; HPLC: high performance liquid chromatography; PCS: pretreated corn stover; TS: total solids.

## Competing interests

The authors declare that they have no competing interests.

## Authors’ contributions

JS executed the pretreatment experiments, analyzed data, and prepared the manuscript along with DJS. JS, DJS, EMK, NJN, MPT, and RTE conceived the work and developed the experimental design. All authors read, revised and approved the final manuscript.

## Supplementary Material

Additional file 1 Supplemental data referred to within the manuscript, including specific component yields in pretreatment and enzymatic hydrolysis, pretreatment mass balance closure and reactor operational conditions. Click here for file
